# Facial Deformities

**Published:** 1900-04

**Authors:** W. E. Davis


ARTICLE IV.

### FACIAL DEFORMITIES.

W. E. DAVIS, D. D. S., CAL.

The title of my paper "Facial Deformities" is, strictly speaking, too comprehensive, and was only adopted owing to the difficulty of obtaining a more sufficiently explicit one. Had it been my intention to thoroughly treat the subject of facial deformities in all various and multiform presentments, I would have attempted an Herculean task, which I am sadly afraid would have necessitated your spending many hours with me, in the end possibly feeling wiser, but undoubtedly sadder men. I therefore will, as far as practicable, speak of those deformities which attract us as dental specialists, leaving those forms that require no dental aid (or rather no aid from the dentist) to their own care. My endeavor will be this evening not much to give you an exhaustive treatise of facial deformities, but to try and impress on you the importance of the work we can accomplish with the plastic surgeon in face operations; and that with our specific help the surgeon can obtain results he is far from gaining now, although I must recognize the fact that, in some cases—but only to a slight extent—our help has been found of advantage by such men, who cannot fail to see the assistance we can give them. What we, as dentists, want to see is that our aid is procured much more generally than is new done, and not in isolated cases, as is at present the condition. At all such times it should be in the power of the dentist to suggest and carry out such treatment so as to leave very little doubt as to prognosis, and only a complete study of facial peculiarities can give us that knowledge which is necessary to the perfect performance of a difficult task, and one calling forth all the mechanical and inventive ability of the operator.

Facial deformities can, for the sake of facility, be classed under the headings of "Congenital" and "Acquired." The deformities coming under the head of the former are those of a hereditary nature, which, by the way, are most resistant to treatment. Further, we have cases where the action of the imagination of the mother upon the unborn child reacts upon it to such an extent that its whole natural contour may be sadly altered; and, again, there is the traumatic deformity, where the child, from the result of a blow, or the application of undue pressure before birth, may bring about a hideous deformity. With these two latter classifications we have little to deal, because the facial contour has been so altered by restricted growth or enlargement of osseous tissue that the most skilful surgeon would be unable to improve on nature's perversion. Those coming under the head of "Acquired" may be subdivided into—

(a) Those deformities resulting from a mechanical action, such as a blow, or

(b) May be the result of disease.

The great question which now arises from our study of the subject is: Are facial deformities on the increase amongst civilized nations? And I unhesitatingly answer:

Yes! (with a large note of exclamation), and there is no doubt in my mind when I give so decided an affirmative to the question. I expect, however, to be assailed for what I consider my emphatic handling. And how do I arrive at the conclusion? I can hear you ask, and what is my chief reason for so doing? It is based on the fact that the original pure type of nations has sadly retrograded, and that national cosmopolitanism is at the root of the evil. Where do we see a nation now-a-days preserving its national type? Nowhere, except a few of our uncivilized peoples. This alteration of specific characteristics is entirely due to the intermarriage of men and women of different nationalities. The mating of the German with the Italian, the Russian with the English, and so on, and nowhere do we see or can we study the peculiar results more so than in America, where emigrants from all nations, representing all types, met together on a common soft and mingled, forming a new nation made up of the multitudinous peculiarities of nearly every people under the sun. And what is the result of this wholesale destruction of the pure type of nations? It is the innocent cause of a large number of facial deformities, much larger than can be easily comprehended in the sunny land of Austria, but which in the United States has grown into a colossal evil; a class of deformity that can cause the possessor to fail to see the delights of society, and when in its most vindictive form, almost ostracises him from the congenial companionship of his fellows. These are the protrusion or retrusion of either jaw, so altering the human face divine that a baboon is a Nenus to it. While on this phase of our subject, I cannot help referring to the able article of Dr. Calvin S. Case, in the "American Text-Book of Operative Dentistry," edited by Dr. E. C. Kirk. It is entitled "The Development of Æsthetic Contours," and, in his opening words, says: In the developmental processes of animal life the teeth have probably been more influential than any of the other organs in shaping the bones of the head, especially in determining the physical characteristics of the physiognomy. The physical shape and structure of the jaws conclusively show the influence that the teeth have exerted in different species in response to nature's laws to propagate that which would best subserve them in the performance of their function. Often the position of the anterior teeth and alveolar process is such as to impress upon the contiguous features, even in respose, certain conditions which vary from a slight imperfection in asthetic contour to a most distressing deformity. The condition of protrusion is generally caused by persons developing the large teeth of one parent and the small jaw of the other, and the logical result is an overcrowding and extended enlargement of the arch, so that, if unattended, it is often most embarrassing. It is not my intention to go into detail regarding the causes of facial deformities from orthodontic complications, for their name is legion and would be a little beside the mark. My principal reason for speaking about these special kinds of deformities is that they may sometimes be lost sight of, and so increase the difficulties the dentist has already to overcome.

I want particularly to impress the fact that now that dental surgery is being recognized as a science, and we as scientific men, it should be our earnest desire, individually and collectively, to propagate thc fact that, in a large number of plastic operations, our assistance as the exponents of—in this case—a mechanical art, can give material help to the surgeon, and that in restoring the face to its normal contour our knowledge of the mouth and its contiguous features would be to an enormous extent to improve that special class of operations at present so much in vogue.

It has been my experience, and I feel certain the experience of all my listeners, that some time or other a surgeon has thanked me for valuable assistance in helping him to obtain a result not possible to him without my aid. This is an old story oft told, but my excuse for dwelling upon it is, that it is of vital importance, it should be ever in our mind, so that an opportunity for promulgating its advantages to the surgeon, and in no small degree to the patient, should never be forgotten. A case in point will help to illustrate my meaning clearer. A young epileptic male, about twenty-five years of age, was brought into the Melbourne Hospital about twelve months ago. He had fallen some distance on to one side of his face. Some faw days after his admission I was sent for, and found that he had fractured the left superior maxilla, deep down in its extent from about the muscular attachment of the depressor ala nasi anteriorly across the canine fossa horizontally to about the region of the maxillary tuberosity posteriorly, and about along its palatal articulation with its fellow of the opposite side. The whole fractured portion was hanging almost loosely in the centre of the mouth. The decision of the surgeon attending was to remove the fractured portion of tissue, and so cause a hideous deformity. There was a great amount of swelling and discoloration, especially around the eye, which was quite closed from view, and I believe the principal reason for removing the fractured bone was from the fact that the patient suffered greatly from epilepsy, and the injury seemed to increase the trouble, so much so that no bandages would remain *in situ* long. I advised trying to restore the fracture and save the removal of any of it, and the case was left in my hands. The patient was chloroformed, and I got the fractured maxilla as much in position as was possible (the principal guide, i. e., the five anterior teeth, were missing), and on taking an impression made an interdental splint, which was attached whilst the patient was again under chloroform. The great difficulty was to keep it in place, for it did not matter how tightly a head bandage was adjusted, during his attacks the splint worked loose and came away. I overcame the difficulty by ligating the splint to the teeth and allowing it to be free from the lower jaw. This plan was entirely successful, and in about two months I removed the splint and found that union had occurred and the parts were quite restored. Externally the contour of the face was perfectly normal.

I could cite many more cases, but I think that "a word to the wise," etc., obtains in this instance. All these things go to show how important our profession can become to the laymen's eyes, and I think that if we steer on these lines, we will have a greater chance of compelling the public to recognize us as scientists, and specialists of a distinct branch of surgery, and of opening a greater vista for our own improvement and research.

Therefore, I say, let every dentist take unto himself the right to instruct his medical friends on this subject, and let those in power see that the rising generation of dentists receive special instruction in this important branch of our profession.

In conclusion, I will read a couple of extreme cases of facial deformities, taken from "Anomalies and Curiosities of Medicine," by Drs. Gould and Pyle," pages 585 and 586.

Injuries destroying great portion of the face and jaw, but not causing death, are seldom seen except on the battlefield, and it is to the military surgery that we must look for the most striking instances of this kind. Ribes mentions a man of thirty-three, who, in the Spanish campaign of 1811, received an injury which carried away the entire body of the lower jaw; half of each ramus, and also mangled in a great degree the neighboring soft parts. He was transported from the field of battle, and despite enormous hemorrhage and suppuration, in two months recovered. At the time of the report the wounded man presented no trade of the inferior maxillary bone, but by carrying the finger along the side of the pharynx in the direction of the superior dental arch, the coronoid apophyses could be recognized and about six lines nearer the temporal extremity the ramus could be discovered. The tongue was missing for about a third of its length, was thicker than natural, and retracted on the hyoid bone. The sub-lingual glands were adherent to under parts of the tongue, and were red and over-developed. The inferior parts of the cheek were cicatrized with the lateral and superior regions of the neck, with the base of the tongue and the hyoid bone. The tongue was free under and in front of the larynx. The patient used a gilded silver plate to fix the tongue so that deglutition could be carried on. He was not able to articulate sounds, but made himself understand through the intervention of this plate, which was fixed to a silver chin, and the chin he used to maintain the tongue plate, to diminish the deformity and to retain the saliva, which was constantly dribbling on the neck.

The same author quotes thc care of a man of fifty who, during the seige of Alexandria of 1801 was struck in the middle of the face obliquely by a cannon ball, from below upwards and from right to left. A part of the right malar bone, the two superior maxillary bones, the nasal bones, the cartilage, the vomer, the middle lemina of the ethmoid, the left maxillary bone, a portion of the left zygomatic arch, and a great part of inferior maxilla were carried away or comminuted, and all the soft parts correspondingly lacerated. Several hours afterwards this solder was counted among the dead, but Larrey, the surgeon-in-chief of the army with his typical vigilance and humanity, remarked that the patient gave signs of life, and that, despite the magnitude of his wound, he did not despair of his recovery. Those portions in which attrition was very great were removed, and the splinters of bone taken out showing an enormous wound. Three months were necessary for cicatrization, but it was not till the capitulation of Marabou, at which place he was wounded, that the patient returned to France. At this time he presented a hideous aspect. There were no signs of nose, nor cartilage separating the entrance of the nostrils, and the vault of the nasal fossa could be easily seen. There was a part of the posterior region of the right superior maxilla—the left was entirely gone—in fact, the man presented an enormous triangular opening in the center of the face. The tongue and larynx were severely involved and the sight in the left eye was lost. This patient continually wore a gilded silver mask, which covered his deformity and rendered articulation a little less difficult. The saliva continually dribbed from the mouth and from the inferior internal portion of his mask, compelling him to carry some substance to receive the dribbings.

Whympter mentions an analogous instance of a gunner who had his whole lower jaw torn away by a shell, but who recovered and used an ingenious contrivance in the shape of a silver mask for remedying the loss of the parts. Steiner mentions a wound from a cannon ball, which carried away the left half of the inferior maxilla, stripping the soft parts as high as the malar, and on the left side of the neck to within one and a-half inches of the clavicle, laying bare the transverse processes of the second and third vertebræ, and exposing the external carotid and most of its branches.

A peculiar case of facial deformity, which I think will be found interesting appeared in this same work, page 697.

A French invalid artillery soldier, from his injuries and a peculiar mask he used to hide them, was known as "*L'homme a la tete de cire*" The *Lancet* gives his history briefly as follows :—

During the Franco-Prussian war he was horribly wounded by the bursting of a Prussian shell. His whole face, including his two eyes were literally blown away. Some scanty remnants of the osseous muscular systems and the skull covered with hair were left. His wounds healed, giving him such a hideous and ghastly appearance that he was virtually ostracised from the sight of his fellows. For his relief a dentist by the name of Delalain constructed a mask, which included a false palate and a set of false teeth.

This apparatus was so perfect that the functions of respiration and mastication were almost completely restored to their former condition, and the man was able to speak distinctly and even play the flute. His sense of smell also returned. He wore two false eyes, simply to fill up the cavities of the orbits, for the parts representing the eyes were closed. The mask was so well adapted to what remained of the real face that it was considered by all one of the finest specimens of the prosthetic art that could be devised, This soldier, whose name was Mareau, was living and in perfect health at the time of the report, his bizarre face, without expression, and his sobriquet, as mentioned, making him an object of great curiosity. He wore the cross of honor, and nothing delighted him more than to talk about the war. To augment his meagre pension he sold a pamphlet containing in detail an account of his injuries and a description of the skilfully devised apparatus, by which his declining life was made endurable. —*Australian Journal of Dentistry.*

